# Treatment Approach to Generalized Severe Periodontitis With the Potential for Additional Tooth Loss: A Case Report

**DOI:** 10.7759/cureus.73336

**Published:** 2024-11-09

**Authors:** Tsvetalina Gerova-Vatsova

**Affiliations:** 1 Department of Periodontology and Dental Implantology, Medical University of Varna, Varna, BGR

**Keywords:** bone loss, periodontal pocket, periodontology, stage iii periodontitis, vertical bone defect

## Abstract

Generalized severe periodontitis, with the potential for additional tooth loss, is one of the most common forms of periodontitis today. Early diagnosis and treatment approaches are of utmost importance. Therapeutic measures must be well thought out and follow a strict sequence. This case report presents the treatment of a patient with stage 3 periodontitis and includes information on both the non-surgical part of the therapy and the surgical intervention in a single area with an existing vertical bone defect. An innovative method of regenerative therapy (guided tissue regeneration with barrier collagen membrane and autogenous platelet-rich plasma) was selected for the treatment of the vertical bone defect in the medial zone of tooth #46.

## Introduction

Periodontitis is an infectious disease whose causative agents cause an inflammatory process involving periodontal components and their subsequent destruction [1,2].

In 2017, Caton et al. [3] presented their idea for classifying periodontal disease and it was accepted by the American Academy of Periodontology and the European Federation of Periodontology. In this classification system, "aggressive periodontitis" and "chronic periodontitis" are grouped into one general category, i.e., "periodontitis." This category is characterized in more detail based on a tabular system concerning staging and grading. Staging is primarily influenced by the extent of the disease at presentation and the complexity of its management. In contrast, grading offers additional insights into the biological characteristics of the disease, including historical analysis of periodontitis progression rates, evaluation of the risk for further progression, examination of potential adverse treatment outcomes, and evaluation of the risk that the disease or its management may adversely impact the patient's overall health [2-4].

Most often, patients with periodontitis seek dental care when pain is present or they have found that one of their teeth is highly mobile. The clinical symptoms of periodontitis are erythema, swelling and bleeding of the gingiva (provoked or not), deposits of dental plaque and calculus present, probing pocket depth >3 mm with clinical attachment loss present, bone resorption present, and, in extremely advanced cases, tooth mobility [5].

Making a prompt and accurate diagnosis is very important to take timely action and minimize the risk of tooth loss. Diagnosis is made through a systematic patient examination protocol, including history, clinical examination, plaque and gingival index assessment, periodontal status, hard tissue examination, radiographic examination, and histological evaluation [6,7]. Patient motivation and corporality and the professional capabilities of the dentist are of utmost importance for achieving long-term therapeutic results.

## Case presentation

In June 2023, a 37-year-old man with complaints of pain, spontaneous bleeding gums, sensation of tooth mobility, and bad breath presented for examination at the University Medical and Dental Center, within the Medical University Varna, Faculty of Dental Medicine.

The patient was first informed in detail about the procedures and gave his written informed consent for the upcoming periodontal procedures.

The patient was in good general health, with no history of allergies, systemic diseases, and regular intake of any type of medication. He reported that he is a non-smoker. No pathological changes were found on extraoral examination. After palpation bilaterally in the submandibular area, single enlarged lymph nodes were found, which were not painful.

**Figure 1:**
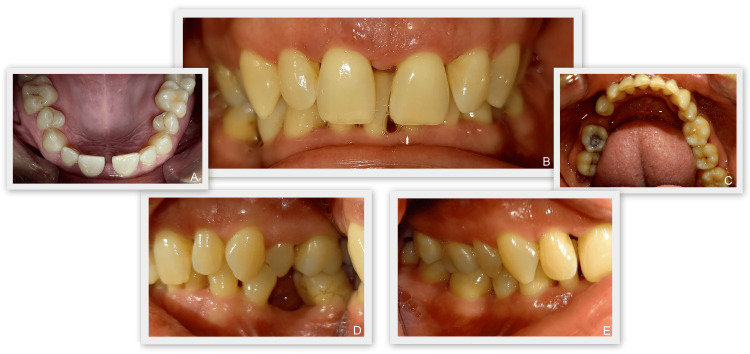
Initial photo documentation. A: Photo documentation of the maxilla. B: Photo documentation (central). C: Photo documentation of the lower jaw. D: Photo documentation (right). E - Photo documentation (left).

Intraoral examination revealed that the color of the gingiva was predominantly pale pink, but areas of hyperemia were present. The interdental papillae were edematous and the gingival margin was thickened. The gingival surface was smooth and shiny. There were areas of established suppuration (tooth #22 medially, between #33 and #34 lingually, tooth #46 medially). Dental plaque and calculus deposits as well as exogenous staining on the tooth surfaces were identified. Gingival recessions covering the entire dentition were present. Carious lesions were found on teeth #16, #37, #38, and #47 (Figure 1).

At the systemic phase of the Ramfjord treatment sequence [6], there were no systemic conditions, systemic diseases, or regular medication intake that were directly etiologically related to periodontal status or that would pose a risk to the patient's general health during treatment. There were also no systemic diseases of the patient that would be risky to the health of the treating staff.

**Figure 2:**
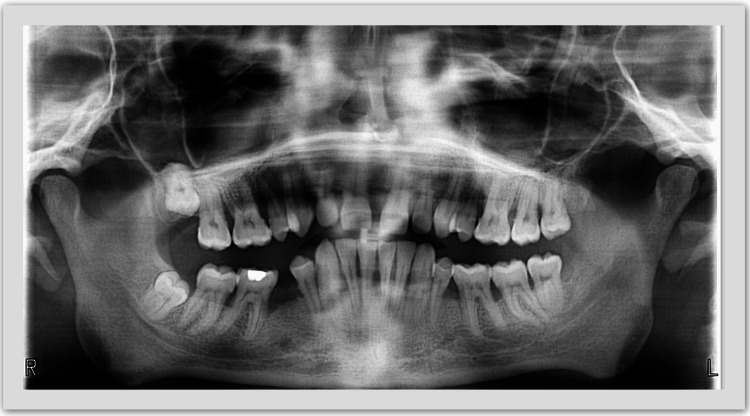
Patient's initial orthopantomography.

After a radiographic examination (orthopantomography) was ordered, the distance from the cementoenamel junction (CEJ) to the alveolar ridge was found to be more than 2 mm in the frontal and distal regions. Lamina corticalis was resorbed, and "unclear" borders were present. The interdental septa in the area of all teeth have lost their characteristic shape and height. Generalized horizontal bone resorption and vertical bone resorption were recorded in the area of tooth #22 (medial), tooth #36 (medial and distal), tooth #37 (distal), and tooth #46 (medial) (Figure 2).

**Figure 3:**
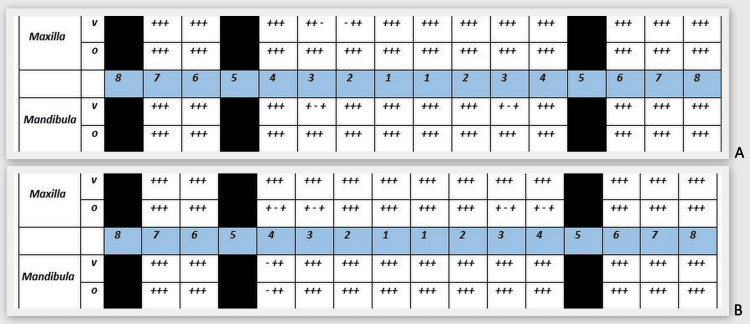
Initial O'Leary's Plaque Index (PI) and Ainamo and Bay's Gingival Index (GI) of the patient. A: PI provides information about the patient's oral hygiene. PI = 152/156*100 = 97.4% - surfaces with the presence of dental plaque. B: GI provides information on the presence and prevalence of gingival inflammation. GI = 150/156*100 = 96.2% - areas with bleeding.

O'Leary's Plaque Index (PI) (Figure 3A) and Ainamo and Bay's Gingival Index (GI) (Figure 3B) were examined during the hygiene phase of the Ramfjord treatment sequence [8]. Ultrasonic supra and subgingival scaling was performed and all tooth surfaces were polished using the LM-ProPower™ CombiLED (LM-Dental, Parainen, Finland).

**Figure 4:**
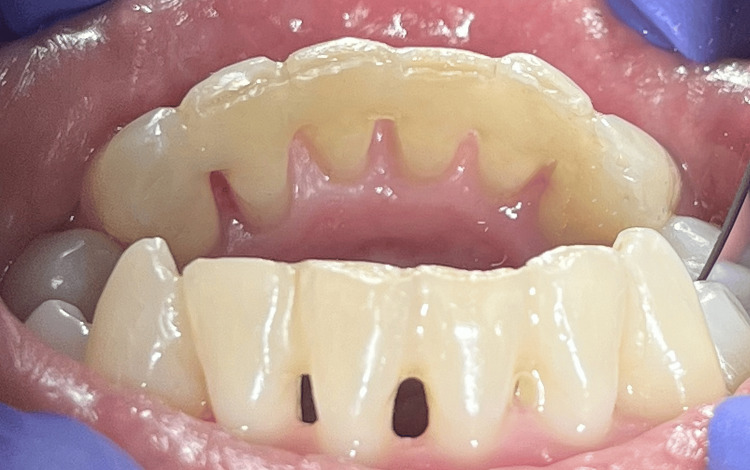
Temporary splinting of lower front teeth with splint fiber and composite.

Temporary splinting with splint fiber and composite was performed in the area of the removable lower front teeth to reduce their overload (Figure 4).

**Figure 5:**
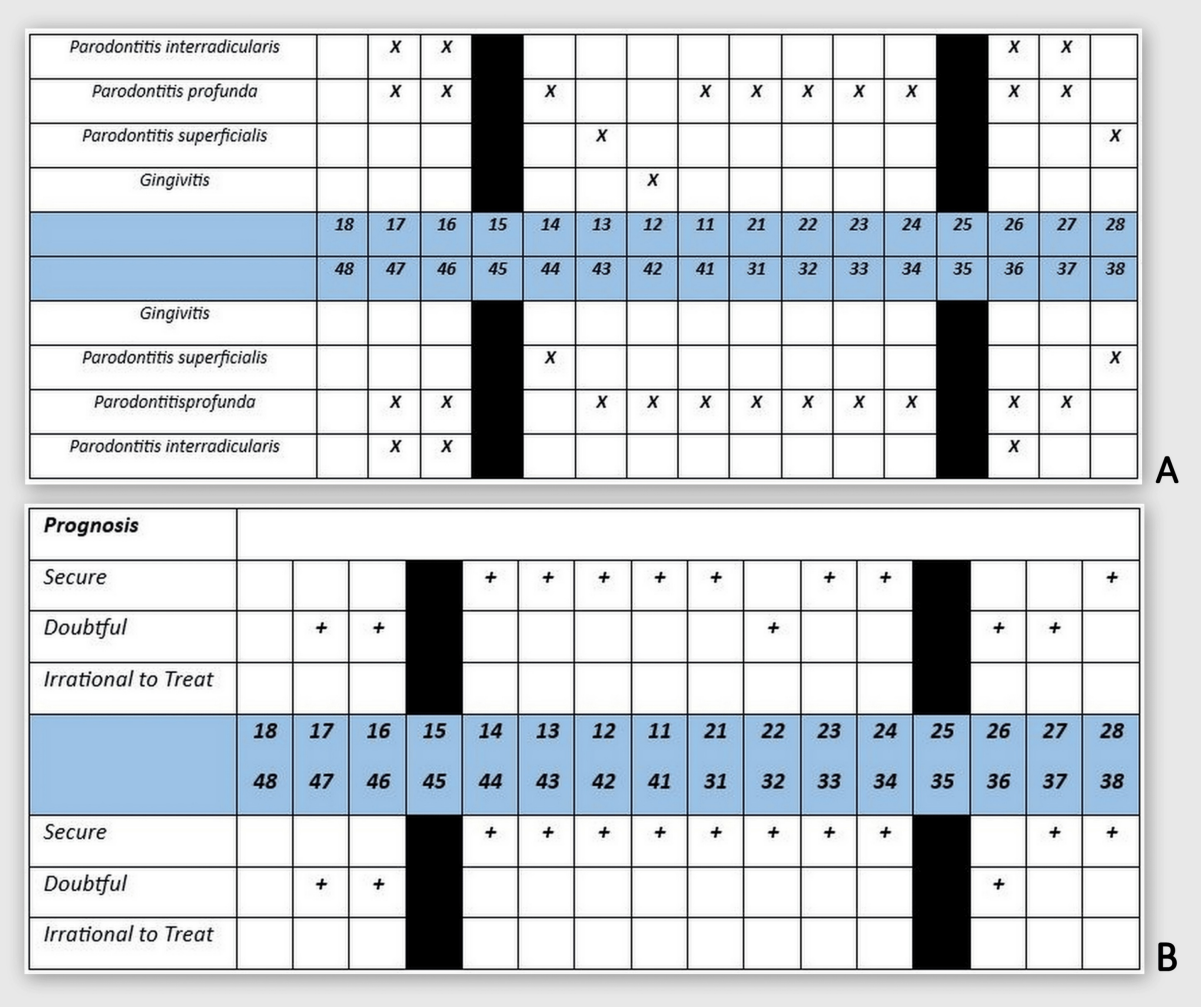
Individual diagnosis and prognosis of each tooth of the dentition. A: Individual diagnosis of each tooth of the dentition. B: Individual prognosis of each tooth of the dentition.

**Figure 6:**
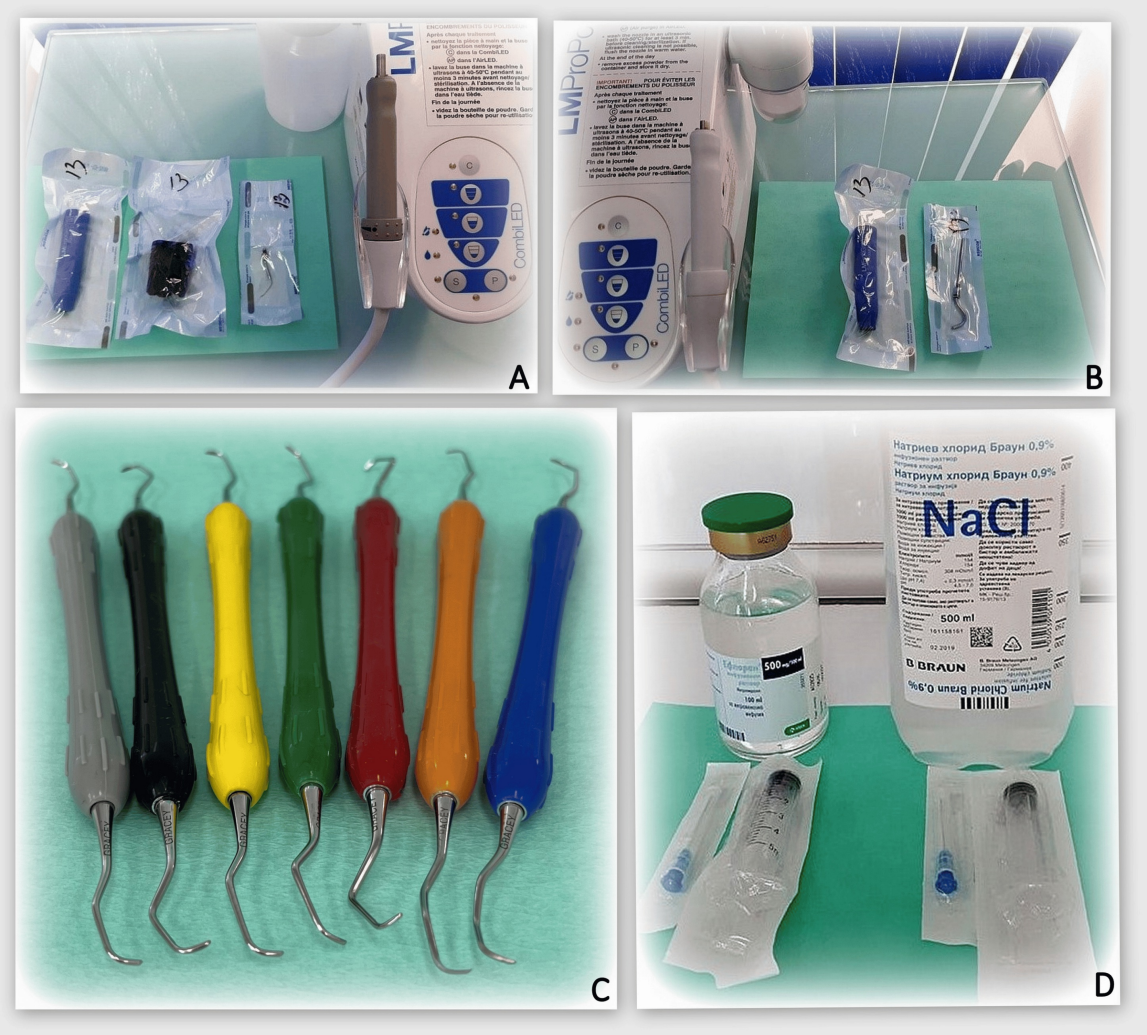
Tools and supplies for performing non-surgical treatment. A: LM-ProPower™ ultrasound device (LM-Dental). B: LM-ProPower™ airflow device (LM-Dental). C: Set of specific Gracey curettes. D: Metronidazole (left) and saline (right).

Two weeks after the ultrasonic cleaning and polishing with the airflow unit, a visit was scheduled during which the current periodontal status was recorded (Figure A1, Appendix). Once the periodontal chart has been completed and an accurate diagnosis and prognosis have been established for each individual tooth (Figure 5), debridement can proceed at the indicated sites. Debridement was performed using specific Gracey curettes with continuous irrigation using saline and metronidazole (Figure 6).

The diagnosis of the whole case was generalized severe periodontitis, with the potential for further tooth loss. The criteria responsible for this are presented in Table 1.

**Table 1:** Criteria for generalized severe periodontitis.

1	According to the severity and complexity of management	Clinical attachment loss, interdental = ≥5 mm (50.7%). Radiographic bone loss - reaches to or passes the middle third of the length of the tooth roots. Tooth #85 lost due to periodontitis. Hypodontia of the second premolars in the area of the 4th quadrant. Maximum probing pocket depth = 10 mm. Vertical bone loss ≥ 3 mm - teeth 22, 36, and 46. Teeth 17, 16, 26, 27, 36, 46, and 47 have class 2 furcation damage. Tooth mobility (grade 1 and 2) and migration.
2	By scope and distribution	Generalized - more than 30% of periodontal units are affected.
3	Evidence or risk of rapid progression, expected response to treatment	Unable to determine the degree of progression (grade A, B, or C) due to lack of radiographs or other documentation from prior.

**Figure 7:**
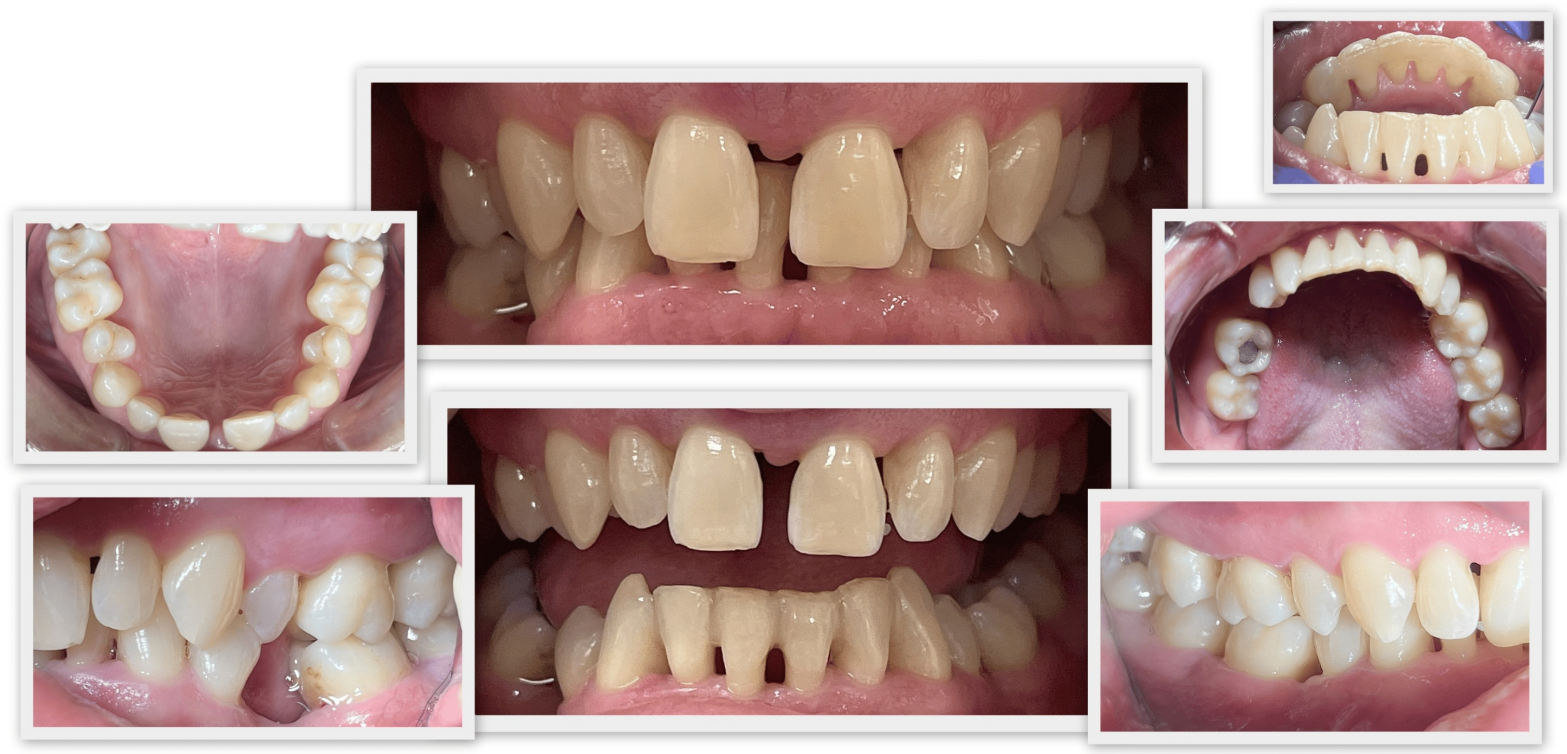
Photo documentation of the "reassessment" phase after the hygiene phase.

**Figure 8:**
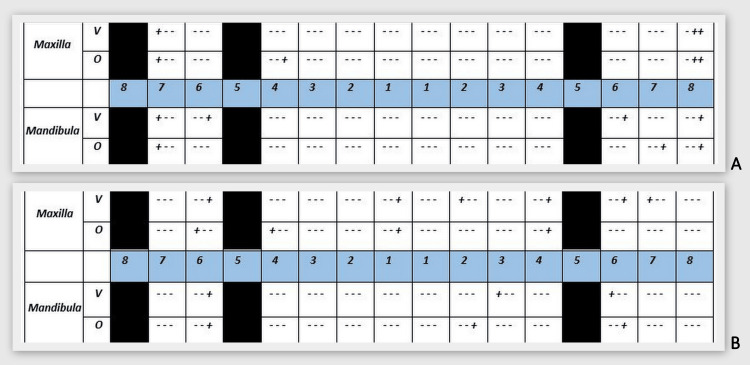
O'Leary's Plaque Index (PI) and Ainamo and Bay's Gingival Index (GI) registered at the "reassessment" stage after the hygiene phase. A: PI = 17/156*100 = 9.0% - surface with the presence of dental plaque. After staining the tooth surface, 9.0% of the surface was found to be covered with plaque, indicating excellent personal plaque control and patient cooperation. Further instructions were given for the hard-to-reach areas. B: GI = 16/156*100 = 10.2% - areas with bleeding.

A reassessment visit was scheduled two months after the debridement was completed. An up-to-date photo documentation was taken (Figure 7), PI (Figure 8A) and GI (Figure 8B) were reassessed, and an up-to-date periodontal status was recorded (Figure A2, Appendix).

There was a reduction in probing depth and a clinical attachment level gain (due to healing processes occurring and a reduction in tissue edema). Residual pockets were identified, some of which are amenable to re-instrumentation: #16, #14, #11, #22, #24, #26, #27, #36, #33, #32, and #46. The results achieved in terms of oral hygiene and gingival status make it possible to plan surgical treatment at the indicated sites.

The clinical results of the non-surgical treatment of this case are summarized in Table 2.

**Table 2:** Comparison of the non-surgical clinical results.

	Hygiene phase	Reassessment after the hygiene phase
PD (average pocket depth)	≈ 5.2 mm	≈ 3.6 mm
CAL (average clinical attachment level loss)	≈ 4.9 mm	≈ 3.7 mm
% of dentition covered by loss of attachment	100.0%	98.7%
	1-2 mm - 30 (19.2%), 3-4 mm - 47 (30.1%), ≥5 mm - 79 (50.7%)	1-2 mm - 45 (28.9%), 3-4 mm- 55 (35.3%), ≥5 mm - 54 (34.6%)

**Figure 9:**
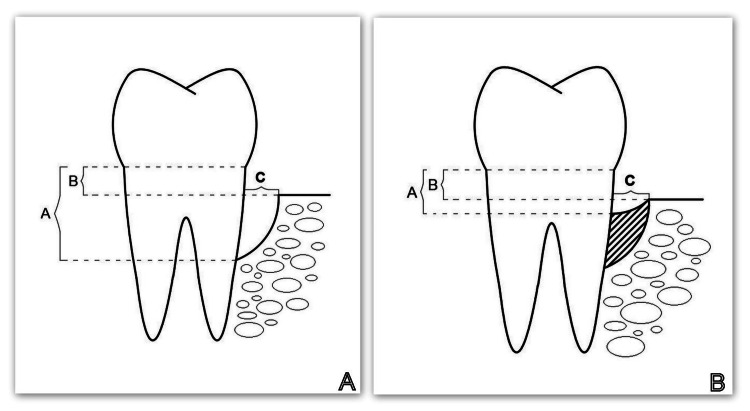
Cone beam computed tomography (CBCT) parameters (A, B, and C). A: CBCT parameters studied before regenerative therapy. B: CBCT parameters studied six months after regenerative therapy. Parameter A: The distance from the cementoenamel junction (CEJ) to the bottom of the bone defect. Parameter B: The distance from the CEJ to the apex of the bone defect. Parameter C: The width of the defect. Image credits: Gerova-Vatsova T.

**Figure 10:**
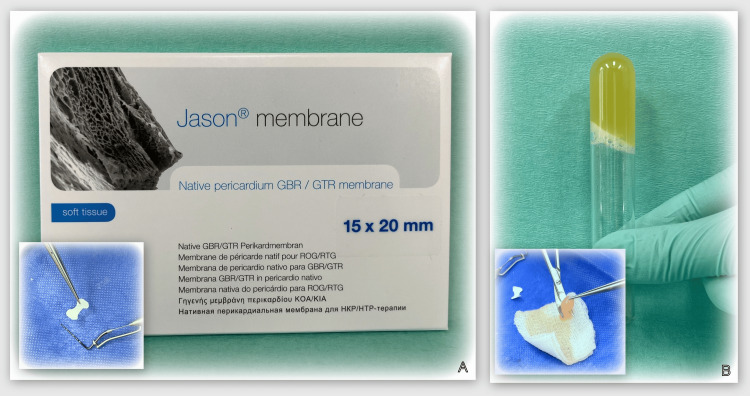
Materials for GTR with barrier membrane and PRP. A: Botiss Jason membrane (Berlin, Germany). B: PRP material after specific processing of the peripheral blood. GTR: guided tissue regeneration; PRP: platelet-rich plasma.

At the time of the re-evaluation visit, the patient was scheduled for a cone beam computed tomography (CBCT) scan in the area of tooth #46. The reason for this (apart from the clinical indications and the vertical defect visible on the orthopantomography) was that the patient reported severe discomfort in the area. Three CBCT parameters (A, B, and C) were recorded, as shown in Figure 9. A visit was planned to perform guided tissue regeneration (GTR) with a barrier collagen membrane and autogenous platelet-rich plasma (PRP) (Figure 10).

**Figure 11:**
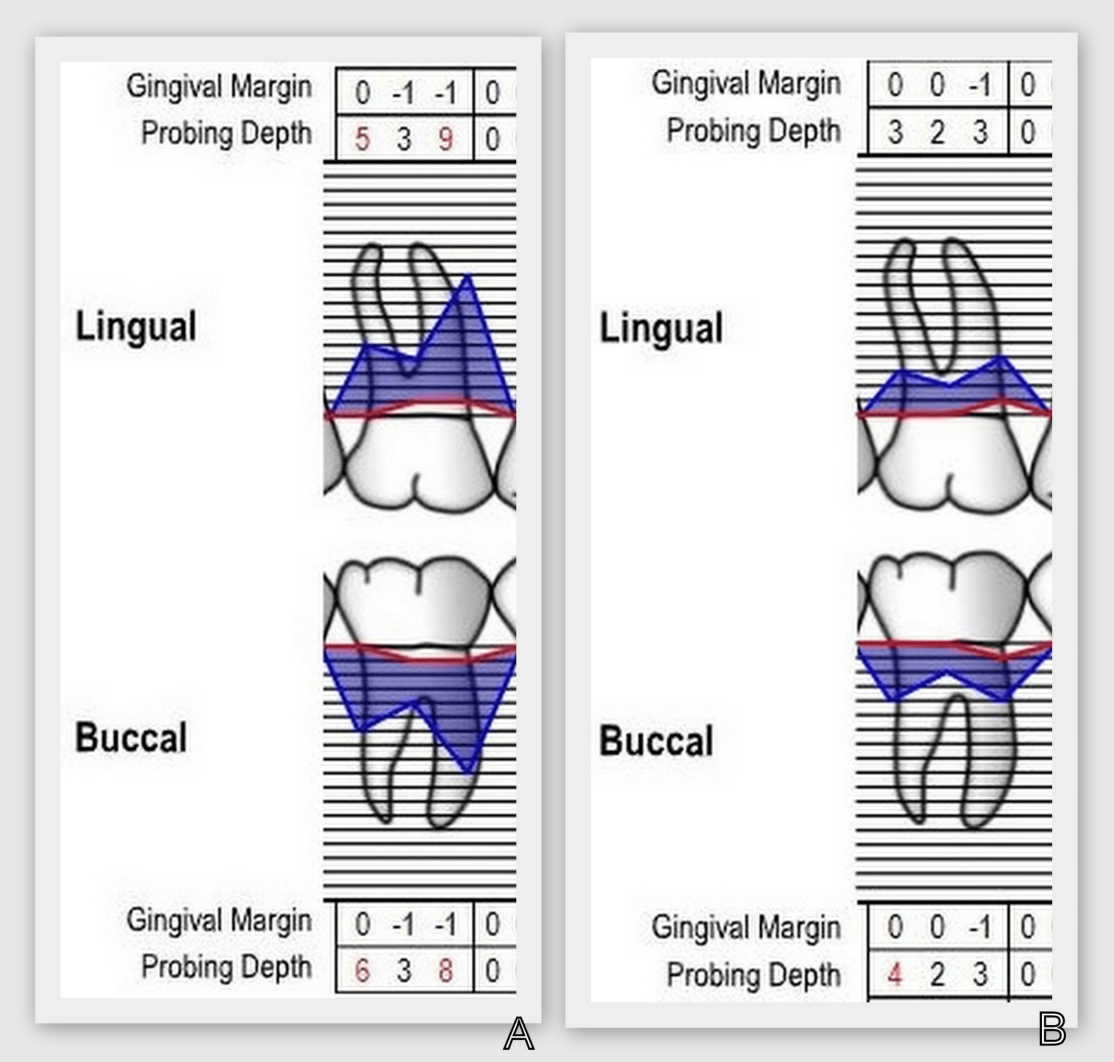
Clinical data before and after regenerative therapy. А: "Reassessment" stage after the hygiene phase. В: Six months after guided tissue regeneration with barrier collagen membrane and platelet-rich plasma.

Clinical data of periodontal status were compared at the "reassessment" stage after the hygiene phase and at the sixth month after GTR with a barrier collagen membrane and PRP (Figure 11).

**Figure 12:**
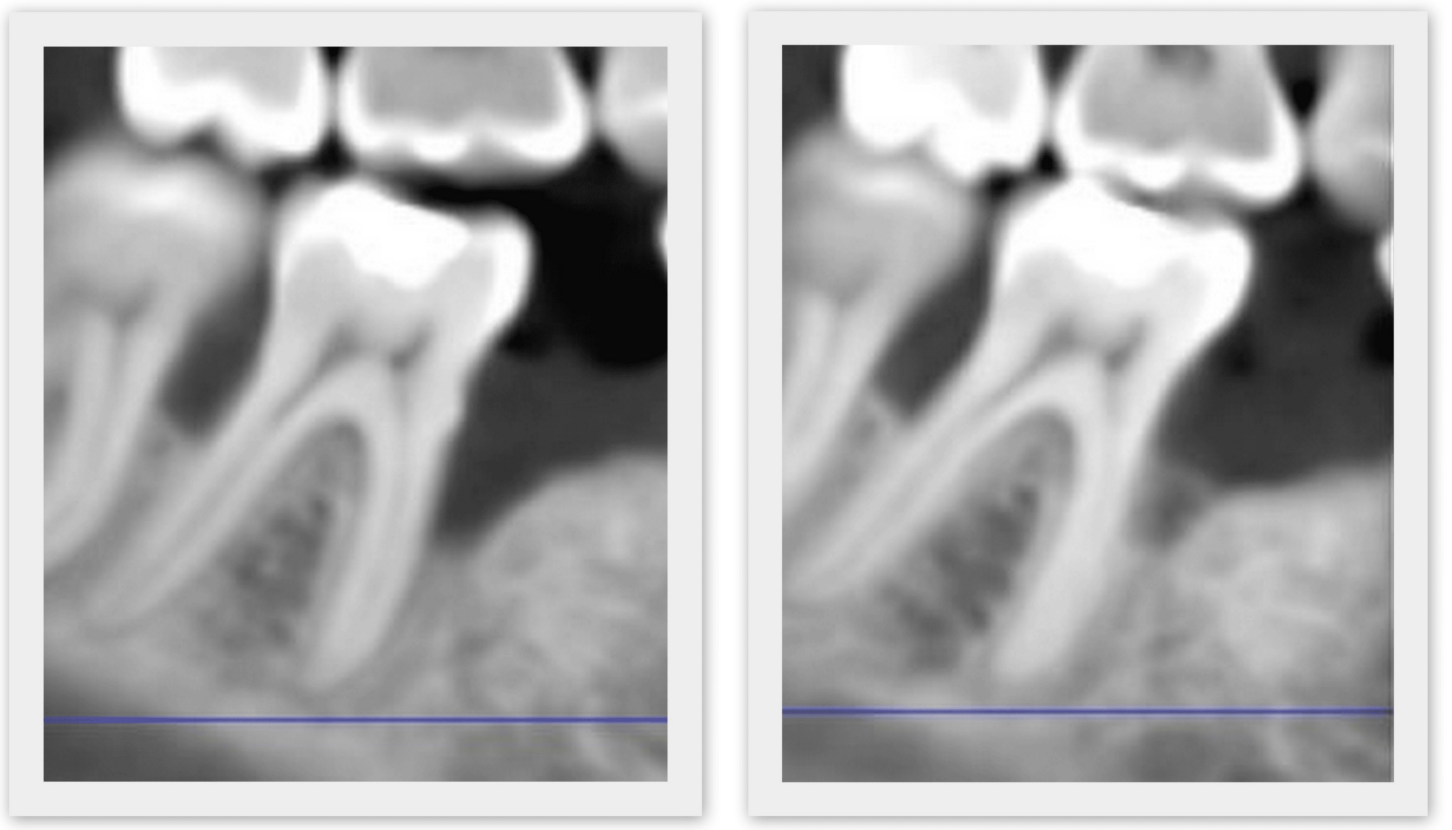
Cone beam computed tomography imaging before and after regenerative therapy.

Six months after the regenerative therapy, a new CBCT study was ordered and the same CBCT indices were measured (Figure 12).

The results of non-surgical therapy in the patient were impressive. Of course, in the area of tooth #46, conventional nonsurgical manipulations alone were not sufficient, which is why regenerative therapy was planned. An innovative method was applied, which has not been described in the literature so far. Comparison of the results of clinical and CBCT parameters immediately before GTR with barrier collagen membrane and PRP (without bone grafts) and six months after are sufficiently indicative and clearly state the potential of this method for the future.

## Discussion

Today, periodontal disease is one of the most common oral diseases. Dentists encounter this diagnosis daily in their practice. Unfortunately, these diseases are often underestimated and neglected in the treatment plans of patients. However, it is an undeniable fact that they are of great social importance among the population. This determines the demand and supply of an effective treatment approach.

Ramfjord's treatment sequence includes four phases: systemic, hygiene, corrective, and maintenance. It is in this treatment approach that the success of non-surgical and surgical periodontal therapy lies today [8-10].

As can be observed in this publication, non-surgical periodontal therapy, with good motivation on the part of the patient and exquisitely performed procedures on the part of the dentist, leads to more than impressive results. Often, surgical treatment is not even necessary in areas where we were convinced it would be necessary before starting nonsurgical therapy [11].

Of course, it is not uncommon to find cases in which nonsurgical periodontal treatment is not sufficient. Fortunately, today we have a palette of different methods (variations of GTR, regenerative therapy with enamel matrix derivative) and materials (barrier membranes, bone repair materials, growth factors, and combinations of the following). All of them are applied with the idea of improving the results achieved in certain areas after nonsurgical treatment [11-13].

In the case presented above, a vertical bone defect was found in the area medial to tooth #46 and indicated for regenerative therapy. The exact size and boundaries of the bone defect were recorded by CBCT to increase the accuracy of the data. CBCT is considered one of the most reliable methods for the diagnosis of bone defects [14]. At this stage, there are many regenerative methods available, and the materials used in these approaches are extremely varied and increasing every day [15-18]. The method chosen for the study is innovative. The goal was to determine whether the established efficacy of barrier collagen membranes over the years, together with the remarkable properties of PRP, would synergize their potential for even better results [19,20].

## Conclusions

For the successful treatment of periodontal disease, it is essential that dentists prepare a preliminary treatment plan for their patients and follow it strictly. The patient's motivation as well as the practitioner's practical skills are invariably linked and play a key role in the success of non-surgical treatment. After analyzing the data of the presented case, we can conclude that the nonsurgical approach, with its impressive results, will always represent a constant in periodontal therapy.

Although today we try to work as non-invasively as possible, sometimes nonsurgical periodontal therapy is not enough. Fortunately, we now have a huge variety of methods and materials available for use in periodontal surgery. Regarding the surgical part of the presented case, it can be concluded that GTR with barrier membrane and PRP is a regenerative method with remarkable capabilities. However, it remains to be seen whether this method will inspire researchers to future studies.

## Figures and Tables

**Figure 13:**
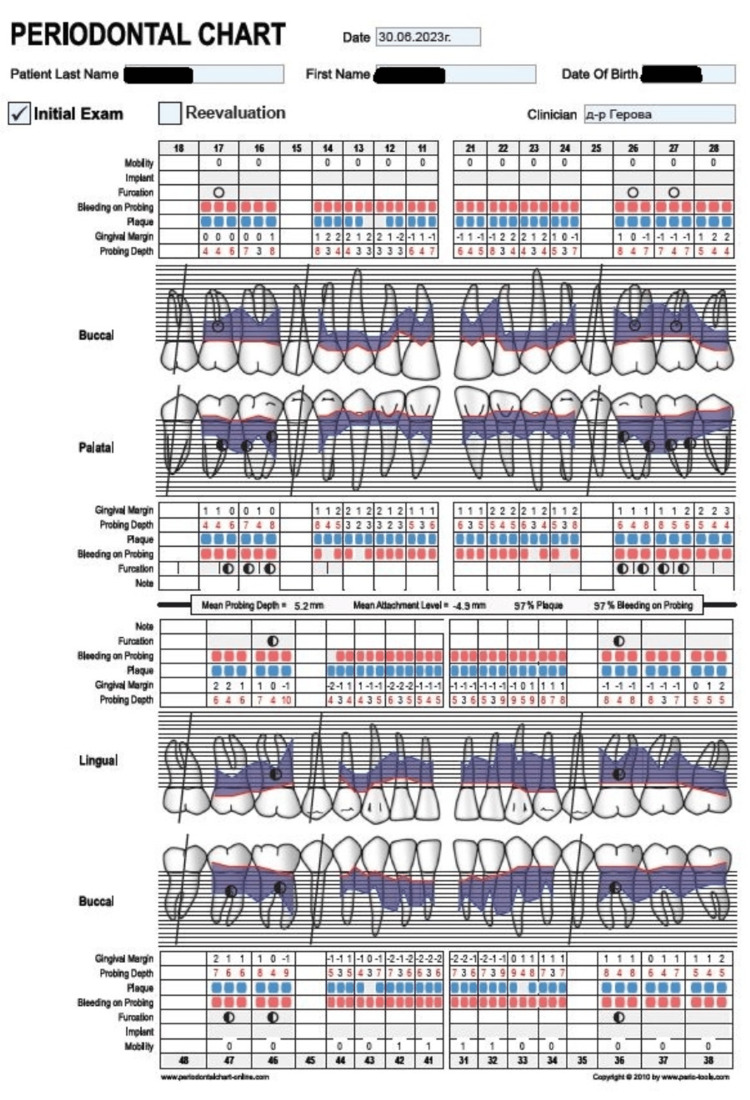
Initial periodontal chart of the patient.

**Figure 14:**
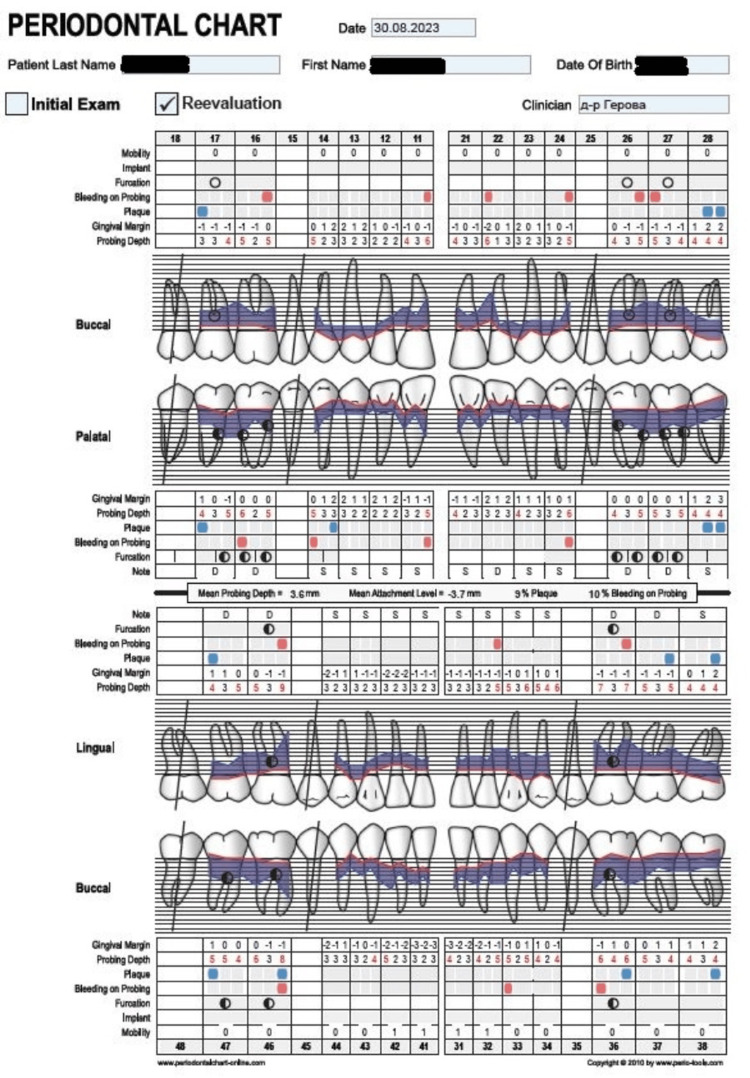
Periodontal chart of the patient at the "reassessment" stage after the hygiene phase.

## Appendices

Figure A1

Figure A2
